# TCOF1 Regulates Tumor Cell Migration Through p53-Dependent Mitochondrial Homeostasis and F-Actin Dynamics

**DOI:** 10.3390/cimb48050447

**Published:** 2026-04-24

**Authors:** Yuanyuan Jiang, Yao Wei, Daikang Yang, Jiali Yao, Qiping Hu, Xiaocui Li

**Affiliations:** 1 Department of Cell Biology and Genetics, School of Pre-Clinical Medicine, Guangxi Medical University, Nanning 530021, China; 2 Key Laboratory of Longevity and Aging-Related Diseases of Chinese Ministry of Education, Guangxi Medical University, Nanning 530021, China; 3 Key Laboratory of Biological Molecular Medicine Research (Guangxi Medical University), Education Department of Guangxi Zhuang Autonomous Region, Nanning 530021, China

**Keywords:** TCOF1, mitochondrial dysfunction, p53, F-actin, STORM

## Abstract

The deficiency of TCOF1 is closely associated with multiple cellular dysfunctions, but its function in mitochondrial homeostasis and cytoskeletal regulation remains unclear. First, our research revealed that TCOF1 deficiency significantly inhibits tumor cell migration, suggesting TCOF1 plays a crucial role in cellular motility. Further studies demonstrated that TCOF1 deficiency disrupts normal F-actin polymerization, compromises cytoskeletal structural integrity, and impairs the dynamic assembly of F-actin, thereby affecting cell morphology and motility functions. Additionally, TCOF1 deficiency leads to mitochondrial dysfunction characterized by aberrant energy metabolism. Mechanistically, TCOF1 deficiency decreased the protein levels of p53, subsequently affecting mitochondrial biogenesis and functional maintenance, suggesting TCOF1 may regulate mitochondrial homeostasis via a p53-dependent pathway. Collectively, our study reveals TCOF1’s role in regulating tumor cell migration by influencing F-actin assembly and the p53-mitochondrial axis, playing a critical role in maintaining cytoskeletal dynamics and energy metabolism.

## 1. Introduction

TCOF1 (Treacle ribosome biogenesis factor 1), encoded by the TCOF1 gene at chromosome locus 5q32–q33.1, is a nucleolar phosphoprotein essential for ribosome biogenesis in eukaryotes. It primarily functions within the cell nucleus, regulating the transcription of ribosomal RNA in eukaryotes [1]. It comprises 28 exons encoding a treacle-phosphorylated protein, a 1411-amino acid nucleochromatin-associated protein that synergizes with upstream binding factor (UBF) to promote ribosomal DNA transcription [2,3,4]. TCOF1 also plays a role in the reactive oxygen species-induced DNA damage repair [5], and its deficiency triggers p53-dependent apoptosis [6,7,8]. TCOF1 was initially identified as the gene responsible for Treacher Collins syndrome, a rare genetic disorder characterized by severe craniofacial malformations [9,10]. To date, the molecular basis of Treacher Collins syndrome, including the specific role of TCOF1 mutations, has been extensively studied [6,10]. In recent years, with the advancement of research, TCOF1 has been found to participate not only in the biosynthesis of ribosomal proteins but also in the regulation of a broader range of cellular processes. By modulating the transcription of nuclear-encoded mitochondrial proteins, TCOF1 links the ribosome biogenesis process in the nucleolus to the energy metabolic homeostasis of mitochondria [11].

Mitochondria are energy-metabolizing organelles widely distributed in eukaryotic cells, playing critical roles including energy production through oxidative phosphorylation, integration of various metabolic pathways, regulation of apoptosis, and maintenance of calcium homeostasis [12,13]. Abnormal mitochondrial function prevents tumor cells from relying on normal oxidative phosphorylation for energy generation, forcing them to switch to inefficient glycolysis [14]. Mitochondria are highly dynamic organelles that undergo continuous remodeling through fusion and fission. Fission plays a role in mitochondrial biogenesis, isolating damaged mitochondrial segments for mitophagy, while fusion promotes the mixing of mitochondrial contents, leading to complementation [15]. As the primary site of oxidative respiration for ATP production, mitochondrial dynamics are implicated in several cellular processes, including the cell cycle, apoptosis, migration, and the generation of reactive oxygen species (ROS) [16]. Mitochondria are semi-autonomous intracellular organelles that are critically important for cellular physiological activities. TCOF1 deficiency leads to mitochondrial dysfunction. For example, TCOF1 can promote the transcription of mitochondrial functional proteins and restore mitochondrial dysfunction in aging cells [11]. In Drosophila models, it has been demonstrated that loss of function in key mitochondrial fission genes leads to mitochondrial network fragmentation and abnormal distribution, accompanied by reduced ATP production and ROS accumulation, thereby triggering synaptic transmission impairment and neuronal functional failure [17,18,19]. Transferred macrophage mitochondria activate downstream signaling pathways in cancer cells via a ROS-dependent mechanism [20].

p53 is a tumor suppressor gene discovered in the 1980s, located within approximately 16–20 kb of genomic DNA on the short arm of human chromosome 17 [21]. It encodes a 53 kDa protein capable of binding to double-stranded or single-stranded DNA to regulate gene transcription [22]. Recent studies have revealed that p53 also functions as a transcription factor regulating energy metabolism signaling pathways, exhibiting effects of reducing glycolysis and enhancing cellular mitochondrial respiration [23]. Its regulation of energy metabolism primarily occurs through inducing TIGAR to decrease glycolysis and enhancing mitochondrial aerobic respiration. p53 deficiency leads to reduced oxygen consumption and a shift in cellular metabolism from aerobic respiration to glycolysis, thereby hindering improvements in aerobic endurance capacity [23,24]. p53 and mitochondria are interconnected through multiple mechanisms. Dysfunction in both pathways frequently occurs concurrently across multiple cardiovascular diseases (CVD). For instance, in the myocardial tissue of patients with dilated cardiomyopathy, upregulation of p53 expression is accompanied by a marked elevation in levels of Bax, a key protein in the mitochondrial apoptosis pathway [25]. p53 can influence mitochondrial respiration by regulating the synthesis of cytochrome c oxidase 2 (SCO2) [26]. Under oxidative stress, p53 can be sequestered within mitochondria to inhibit apoptosis [27]. p53 can protect mtDNA by interacting with TFAM [28]. Moreover, p53 promotes mitochondrial apoptosis by either regulating the expression of multiple B-cell lymphoma 2 (BCL-2) family proteins through transcription or by engaging in physical interactions with them [29].

The cytoskeleton is composed of three major filament systems: microtubules, intermediate filaments, and microfilaments [30]. Among these, microfilaments consist of actin, the most abundant protein complex in eukaryotic cells. Its dynamic changes play crucial roles in multiple processes, including cell migration, cytokinesis, vesicular transport, and phagocytosis [31,32]. F-actin is the polymerized form of actin, assembled from globular(G)-actin into a dynamic double-helix fiber structure. It serves as a core component of the cytoskeleton, participating in various physiological processes such as muscle contraction, cell motility, and shape maintenance. Crosstalk between actin and the microtubule cytoskeleton is critical for numerous cellular processes, including cell division, migration, and intracellular transport [33,34]. This study aims to elucidate how TCOF1 regulates tumor cell migration through p53-dependent mitochondrial homeostasis mechanisms and F-actin dynamics, thereby providing new insights into the role of TCOF1 in developmental abnormalities and tumorigenesis.

## 2. Materials and Methods

### 2.1. Cell Culture and Gene Silencing

U2OS cells were obtained from the American Type Culture Collection (Manassas, VA, USA). Cells were cultured in DMEM supplemented with 10% fetal bovine serum and 1% penicillin/streptomycin. Cultures were maintained at 37 °C in a 5% CO_2_ cell culture incubator. Mycoplasma was regularly examined during cell culturing, and no contamination occurred during this study. siRNAs were transfected into target cells using Lipofectamine^®^ RNAiMAX transfection reagent (Invitrogen, Carlsbad, CA, USA) according to the manufacturer’s protocol. The sequences of the siRNAs used in this study are listed in Table 1.

### 2.2. Immunofluorescence (IF) Analysis

Cells cultured on glass coverslips were first immobilized with 4% paraformaldehyde for 15 min, permeabilized with 0.2% Triton X-100 (The Dow Chemical Company, Shanghai, China) for 10 min. Non-specific binding sites were blocked with 5% goat serum for a duration of 1 h. Subsequently, the fixed specimens were incubated with the corresponding primary antibodies overnight at 4 °C, after which they were exposed to fluorescence-conjugated secondary antibodies for 1 h at room temperature (20–25 °C). Nuclei were counterstained with DAPI, and images were captured with a Zeiss microscope (Zeiss, Graz, Austria).

### 2.3. Stochastic Optical Reconstruction Microscopy (STORM) Analysis

Super-resolution images were acquired with a STORM system established by NBI (NanoBioImaging Ltd., Hongkong, China), specifically designed for dual-channel imaging of AlexaFluor 647- and AlexaFluor 750-immunolabeled samples [35]. Cells were grown on coverslips immobilized with polystyrene particles (Spherotech, Lake Forest, IL, USA), fixed with cold methanol on ice for 10 min, then incubated at room temperature (20–25 °C) in 0.2% Triton X-100 for 10 min. After washing, coverslips were incubated with blocking solution at room temperature (20–25 °C) for 1 h. Excess blocking solution was removed by washing with PBS, followed by incubation with dye-conjugated primary antibody in blocking solution at room temperature for 1 h to complete specific labeling. Coverslips were washed, incubated in 4% paraformaldehyde solution for 10 min for fixation, and then thoroughly rinsed with PBS. Each super-resolution image was reconstructed by Fiji from a movie containing 10,000 frames. During acquisition, the dye molecules in the TCEP-containing imaging buffer cycled repeatedly between dark and bright states.

### 2.4. RT-qPCR

Total RNA extraction was conducted utilizing RNAiso Plus (Takara, Shiga, Japan), and complementary DNA (cDNA) synthesis was performed with the PrimeScript II First-Strand cDNA Synthesis Kit (Takara, Shiga, Japan), following the protocols provided by the manufacturer. qPCR assays were executed using the 2× Universal SYBR qPCRMix (Biosharp, Hefei, China). GAPDH was used for normalization. RT-qPCR primers are listed in Table 2.

### 2.5. Western Blotting

Transfected cells were harvested and lysed in RIPA buffer on ice for 1 h. Protein concentrations were determined, and samples were prepared for analysis. Protein samples were separated by SDS-PAGE electrophoresis, transferred for 1.5 h, blocked with 5% nonfat milk in TBST at room temperature for 1 h, incubated with primary antibody overnight at 4 °C, washed three times with TBST, incubated with secondary antibody at room temperature for 1 h, washed three times with TBST, developed using ECL chemiluminescence, and analyzed for band intensity using ImageJ software (v1.53t).

### 2.6. Wound Healing Assay

Draw three horizontal lines on the back of a 6-well plate using a colored marker pen. Collect the cells and seed 1 × 10^6^ cells onto the 6-well plate. Incubate in a cell culture incubator at 37 °C and 5% CO_2_. On the following day, when cell confluence reaches 90%, use a sterile 200 μL pipette tip to draw three evenly spaced lines perpendicular to the well plate. Carefully aspirate the original culture medium, wash twice with 2 mL of PBS to remove residual cell debris, then place the cells in serum-free medium. Under an inverted microscope, capture an initial image of the scratched area at a fixed magnification (designated as 0 h). Return the culture plate to the cell culture incubator (37 °C, 5% CO_2_). Capture representative images at 24 h, 48 h, and 72 h after scratching, ensuring that the same field of view is recorded at each time point. Analyze these images using software and calculate the scratch healing rate according to the formula: (W0 h − W24 h)/W0 h × 100% (where W represents the width of the scratch). All experiments were repeated three times.

### 2.7. Phalloidin Staining Assay

Discard the culture medium, and wash the cells twice with PBS. Fix the cells with 4% paraformaldehyde for 15 min at room temperature. Wash three times with PBS containing 0.1% Triton X-100, each for 5 min. Incubate with phalloidin diluted in PBS containing 1% BSA and 0.1% Triton X-100 at room temperature in the dark for 1 h. Wash three times with PBS. Add 100 ng/mL DAPI to the appropriate position on the slide and mount.

### 2.8. Cell Treatment

U2OS cells were seeded in 24-well plates lined with coverslips. When cell confluence reached 30–40%, the cells were transfected with the negative control NC, siTCOF1-1, or siTCOF1-2, respectively. Twenty-four hours after transfection, the medium was replaced with complete medium containing 0.5 μM or 1 μM rotenone (MCE), or an equal volume of DMSO (solvent control), and treatment was continued for 48 h. Subsequently, the cells were washed three times with PBS (5 min each time), fixed with 4% paraformaldehyde at room temperature for 20 min, washed three times with PBS (5 min each time), and then stained with phalloidin following the procedure described in Section 2.7. Phalloidin Staining Assay: Under a laser confocal microscope (Nikon Corporation, Tokyo, Japan), acquire DAPI (blue) and phalloidin (red) channels as well as merged images using a 100× oil immersion objective to observe changes in F-actin morphology and cell polarity.

### 2.9. RNA Sequencing

U2OS cells from the TCOF1 deficiency group and negative control group were collected for RNA sequencing (*n* = 3), conducted by Beijing Tsingke Biotechnology Co., Ltd. (Beijing, China) following Illumina (San Diego, CA, USA) protocols. Libraries were prepared with the Illumina^®^ Stranded mRNA Prep Ligation Kit (Illumina, San Diego, CA, USA) using 1 μg total RNA per sample: mRNA was enriched via oligo (dT)-coupled magnetic beads, fragmented into ~300 bp fragments, reverse-transcribed into double-stranded cDNA, and subjected to end repair, phosphorylation, 3′-A tailing, Y-shaped adapter ligation, purification, size selection, and PCR amplification. Libraries were quantified by Qubit 4.0 and qualified by Agilent 5300 Bioanalyzer (Agilent, Waldbronn, Germany) before paired-end sequencing (2 × 150 bp) on Illumina NovaSeq X Plus. Raw data were quality-controlled with SOAPnuke (v1.5.2), and clean reads were aligned to the human GRCh38/hg38 genome via HISAT2 (v2.0.4). Gene expression (TPM) was quantified using Bowtie2 (v2.2.5) and StringTie (v2.1.2)/RSEM (http://deweylab.github.io/RSEM/, accessed on 20 April 2026). DEGs were identified by DESeq2 (v1.4.5) (FDR ≤ 0.05, |log2FC| ≥ 1). KEGG and GO enrichment analyses were performed with KOBAS (http://kobas.cbi.pku.edu.cn, accessed on 20 April 2026) and Goatools (https://github.com/tanghaibao/goatools, accessed on 20 April 2026), respectively (corrected *p* ≤ 0.05).

### 2.10. Transwell Cell Migration Assay

The assay was conducted using Transwell chambers with an 8 μm pore size. Following transfection, U2OS cells from each group were harvested and resuspended in serum-free DMEM to form a single-cell suspension. Complete DMEM medium containing 10% fetal bovine serum was added to the lower chamber. Subsequently, 5 × 10^4^ cells in 200 μL of serum-free DMEM medium were seeded into the upper chamber. The culture plates were incubated at 37 °C in a 5% CO_2_ incubator for 24 h. Then, the plates were fixed at room temperature for 20 min, stained with crystal violet for 20 min, and random fields were photographed under an inverted microscope. The experiment comprised three biological replicates. Cell migration to the lower chamber was quantified using ImageJ software. Data are presented as mean ± standard deviation (x ± s), with statistical analysis comparing migration capacity differences between groups.

### 2.11. Statistical Analysis

Statistical analysis was performed using GraphPad Prism 9.5. For bioinformatics data and experimental results, parametric *t*-tests were used to compare differences between groups for normally distributed data, while nonparametric rank-sum tests were applied for skewed distributions. Statistical significance was determined at * *p* < 0.05; ** *p* < 0.01; *** *p* < 0.001; **** *p* < 0.0001.

## 3. Results

### 3.1. TCOF1 Deficiency Inhibits Tumor Cell Migration

**Figure 1:**
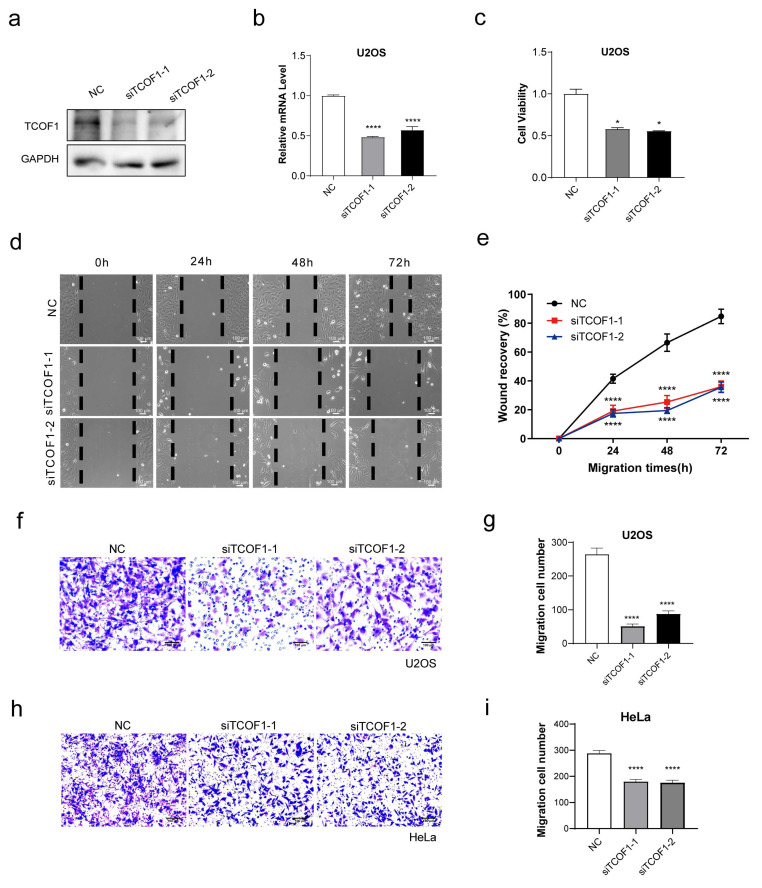
TCOF1 deficiency inhibits U2OS cell migration. (**a**) The knockdown efficiency of TCOF1 at the protein level in U2OS cells was validated by Western blotting analysis. (**b**) The knockdown efficiency of TCOF1 at the mRNA level in U2OS cells was validated by RT-qPCR. (**c**) Effect of TCOF1 deficiency on the viability of U2OS cells. (**d**) Wound healing assay to evaluate the effect of TCOF1 deficiency on U2OS cell migration (10×). (**e**) Quantification of (**d**). (**f**) The effect of TCOF1 deficiency on the migratory capacity of U2OS cells was evaluated using a Transwell cell migration assay. (**g**) Quantification of (**f**). (**h**) The effect of TCOF1 deficiency on the migratory capacity of HeLa cells was evaluated using a Transwell cell migration assay. (**i**) Quantification of (**h**). All values are the average ± SEM of three independent experiments. The unpaired Student’s two-tailed *t*-test was used to determine the statistical significance (* *p* < 0.05; **** *p* < 0.0001). All values are the average ± SEM of three independent experiments.

The migration and invasion capabilities of cancer cells are key indicators for assessing their metastatic potential. To investigate the role of TCOF1 in this process, we performed TCOF1 deficiency experiments in human osteosarcoma U2OS cells. The knockdown efficiency was validated at both the protein and mRNA levels via Western blotting and q-PCR, respectively (Figure 1a,b). TCOF1 depletion decreased the cell viability (Figure 1c). To further investigate TCOF1’s impact on cell migration, we performed wound healing assays and a Transwell migration assay. TCOF1 deficiency markedly inhibited the migratory capacity of U2OS cells (Figure 1d–i). These results collectively indicate that TCOF1 promotes tumor cell migration.

### 3.2. TCOF1 Deficiency Affects F-Actin Assembly

**Figure 2:**
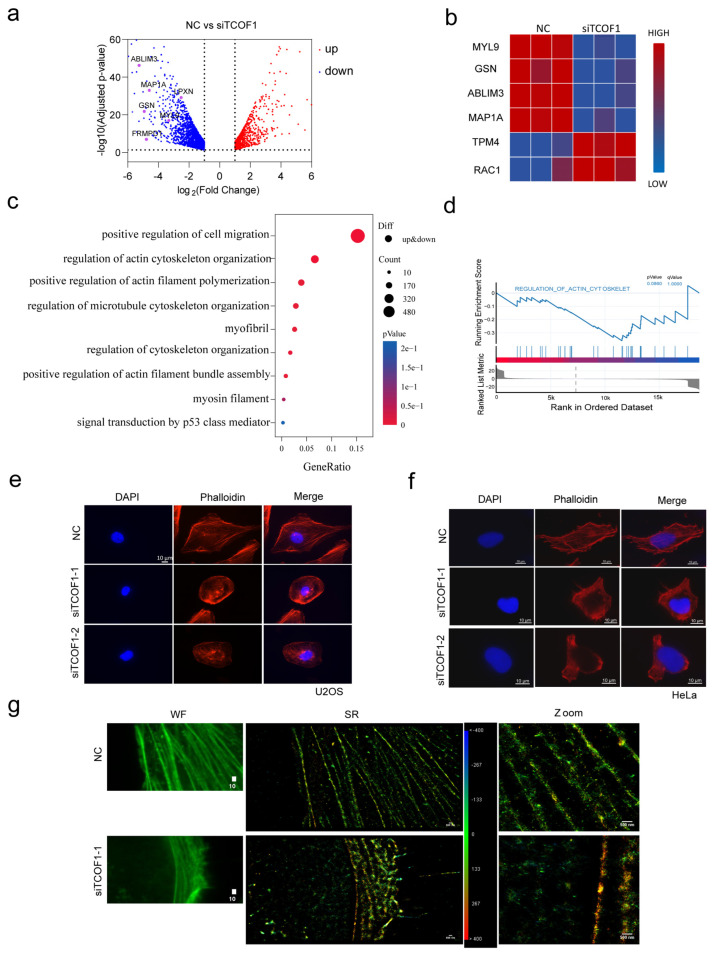
TCOF1 deficiency impairs F-actin formation. (**a**) Volcano plot of differentially expressed genes after TCOF1 deficiency. (**b**) Gene expression heatmap: Differential expression levels of core genes in the actin cytoskeleton regulation pathway between NC and siTCOF1. (**c**) GO gene enrichment analysis bubble plot of differentially expressed genes after TCOF1 deficiency. (**d**) GSEA in TCOF1 deficiency, showing regulation of actin cytoskeleton. (**e**) Phalloidin-labeled F-actin visualization of cytoskeletal changes in U2OS cells. Phalloidin-labeled actin appears as red; DAPI appears as blue. Scale bars: 10 μm. (**f**) Observing changes in the F-actin cytoskeleton following TCOF1 deficiency in HeLa cells. Scale bars: 10 μm. (**g**) Visualization of F-actin using STORM, observation of F-actin under widefield and STORM conditions. Scale bars: wide field 10 μm, SR (super resolution) 500 nm. All values are the average ± SEM of three independent experiments.

From the experimental results above, we can clearly observe that the migration ability of tumor cells significantly decreased, which was associated with TCOF1 deficiency. This observation suggests that TCOF1 may potentially support the migration function of tumor cells. To investigate the underlying molecular mechanisms of this phenomenon, we employed RNA-Seq to analyze genes linked to TCOF1 deficiency. The results revealed that 2035 genes were downregulated following TCOF1 suppression, including several actin pathway-related genes, such as MYL9, LPXN, GSN, FRMPD1, ABLIM3, and MAP1A (Figure 2a). A heatmap was generated using core genes from the actin cytoskeleton regulation pathway (Figure 2b). This heatmap revealed that following TCOF1 deficiency, expression levels of core genes within these pathways exhibited widespread transcriptional alterations. These findings suggest that there may be a link between TCOF1 and the regulation of the actin cytoskeleton. The Gene Ontology analysis of downregulated genes showed that several biology processes related to the cytoskeleton and F-actin were significantly enriched, in addition to the regulation of cell migration (Figure 2c,d). TCOF1 may be involved in the regulatory network of the actin cytoskeleton, but its specific regulatory mechanism remains to be further elucidated. To test this hypothesis, we performed phalloidin staining experiments using phalloidin, a mycotoxin extracted from toxic fungi that specifically binds to filamentous actin [36]. Actin in the negative control (NC) group cells exhibited a relatively extended and normal distribution pattern, indicating that actin structure remains relatively stable under normal or control conditions. Compared to the NC group, the TCOF1 deficiency group exhibited significant changes in actin distribution. F-actin assembled into circular bundles along the cell periphery, accompanied by reduced cytoskeletal polarity (Figure 2e). Similarly, disruption of F-actin assembly was observed following TCOF1 knockdown in HeLa cells (Figure 2f). These results suggest that TCOF1 regulates actin assembly and distribution. Simultaneously, nanoscale-resolution STORM was employed to observe precise changes in F-actin arrangement in U2OS cells (Figure 2g). Altogether, these results demonstrated that TCOF1 deficiency reduced cytoskeletal polarity, impairing F-actin formation.

### 3.3. TCOF1 Deficiency Impairs Mitochondrial Function

**Figure 3:**
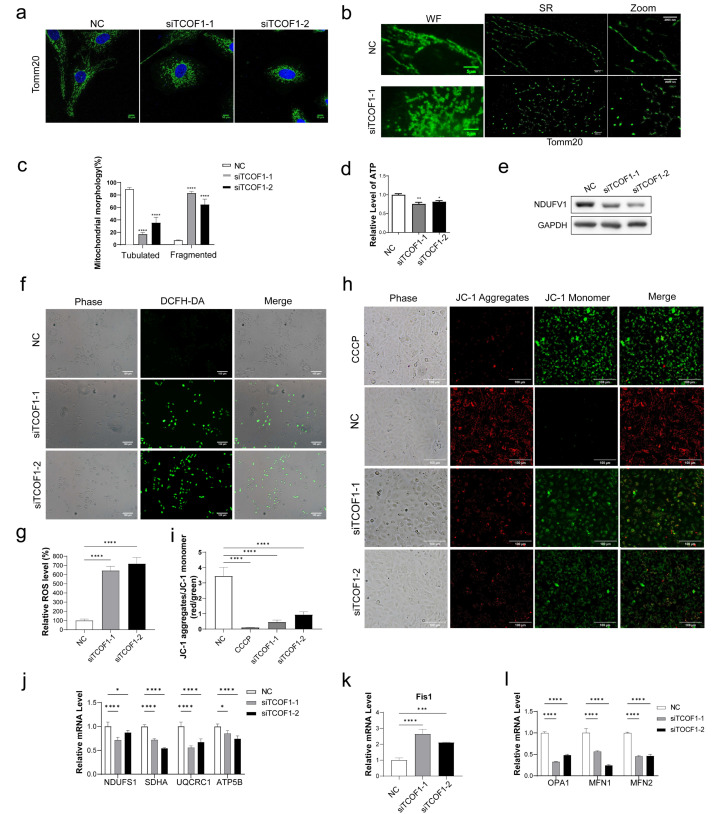
TCOF1 deficiency impairs mitochondrial function. (**a**) Immunofluorescence images showing mitochondrial morphology changes in U2OS cells after TCOF1 deficiency. Mitochondrial outer membrane labeled with Tomm20 (green), nuclei stained with DAPI (blue) (100×). Scale bars: 10 μm. (**b**) Mitochondria visualized using STORM. Mitochondria observed under wide-field and super-resolution conditions. Scale bar: 5 μm (wide field), 2000 nm (SR). (**c**) Quantification of panel (**a**). (**d**) ATP production in U2OS cells following TCOF1 deficiency was detected using an ATP assay kit. (**e**) Effect of TCOF1 deficiency on the protein level of NDUFV1 in U2OS cells. (**f**) Fluorescence images of reactive oxygen species (ROS) in U2OS cells loaded with DCFH-DA probe under NC, siTCOF1-1, and siTCOF1-2 conditions. (**g**) Quantification of panel (**f**). (**h**) Fluorescence microscopy images of JC-1 staining in the indicated groups (Scale bar, 100 μm). CCCP-treated cells served as a positive control for mitochondrial depolarization. U2OS cells were transfected with negative control or siTCOF1-1/2 siRNA, respectively. Red fluorescence (JC-1 Aggregates) indicates intact ΔΨm, whereas green fluorescence (JC-1 Monomer) indicates loss of ΔΨm. Scale bars: 10 μm. (**i**) Quantification of panel (**h**). (**j**) Relative mRNA levels of mitochondrial respiratory chain complex genes NDUFS1, SDHA, UQCRC1, and ATP5B in U2OS cells after TCOF1 knockdown. (**k**) Relative mRNA level of mitochondrial fission-related gene Fis1 in U2OS cells after TCOF1 knockdown. (**l**) Relative mRNA levels of mitochondrial fusion-related genes OPA1, MFN1, and MFN2 in U2OS cells after TCOF1 knockdown. All values are the mean ± SEM of three independent experiments. The unpaired Student’s two-tailed *t*-test was used to determine the statistical significance. All values are the average ± SEM of three independent experiments. (* *p* < 0.05, ** *p* < 0.01, *** *p* < 0.001, **** *p* < 0.0001).

Interactions exist between the cytoskeleton and mitochondria [37], and mitochondrial fission depends on dynamin-related protein 1 and F-actin: when F-actin is disrupted, mitochondrial fission is impaired [38]. Using immunofluorescence (IF) staining combined with STORM technology, we observed that TCOF1 deficiency affects mitochondrial morphology in U2OS cells. In the NC group, mitochondria exhibited a rich variety of morphological features, primarily characterized by an extensive network of wavy tubules. These tubules were distributed roughly radially throughout the cytoplasm. The cells contained almost no spherical mitochondria, or only a very small number, replaced by numerous elongated mitochondrial tubules. In stark contrast, TCOF1-deficient cells exhibited a pronounced mitochondrial fragmentation phenotype. Mitochondria predominantly adopted spherical or elliptical morphologies, with only extremely short tubules visible or appearing as uniformly sized microvesicles, indicating a significant increase in overall fragmentation (Figure 3a–c). Next, we investigated whether TCOF1 deficiency affects ATP production in U2OS cells. Compared to the NC group, TCOF1-knockdown cells exhibited reduced ATP production (Figure 3d). Knocking down TCOF1 resulted in downregulation of the NDUFV1 protein, a mitochondrial respiratory chain gene (Figure 3e). To further validate the impact of TCOF1 knockdown on mitochondrial function, we assessed intracellular reactive oxygen species (ROS) levels. The control group showed almost no fluorescence and low ROS levels, while the TCOF1-depleted group showed high fluorescence intensity, indicating high ROS levels (Figure 3f,g and Figure S1a,b). We used JC-1 staining to assess changes in mitochondrial membrane potential. The results showed that, compared with the NC group, the JC-1 aggregate-to-monomer ratio was significantly decreased in the TCOF1 knockdown group, accompanied by reduced red fluorescence and increased green fluorescence (Figure 3h,i and Figure S1c,d). These findings indicate that TCOF1 deficiency leads to a decrease in mitochondrial membrane potential. We also examined the transcriptional levels of genes encoding subunits I–V of the mitochondrial respiratory chain to investigate whether TCOF1 deficiency affects the assembly and function of these complexes. Compared to the control group, TCOF1 deficiency led to decreased mRNA levels for mitochondrial respiratory chain genes NDUFS1, SDHA, UQCRC1, and ATP5B (Figure 3j). To investigate whether TCOF1 deficiency disrupts the dynamic equilibrium between mitochondrial fission and fusion, leading to abnormal mitochondrial morphology, we also examined mRNA expression levels of key fission/fusion genes. The mRNA level of the mitochondrial fission gene Fis1 was increased (Figure 3k), while the mRNA levels of the mitochondrial fusion genes MFN1, MFN2, and OPA1 were decreased (Figure 3l). These results collectively indicate that TCOF1 deficiency impairs mitochondrial function.

### 3.4. TCOF1 Maintains Mitochondrial Stability by Regulating p53

**Figure 4:**
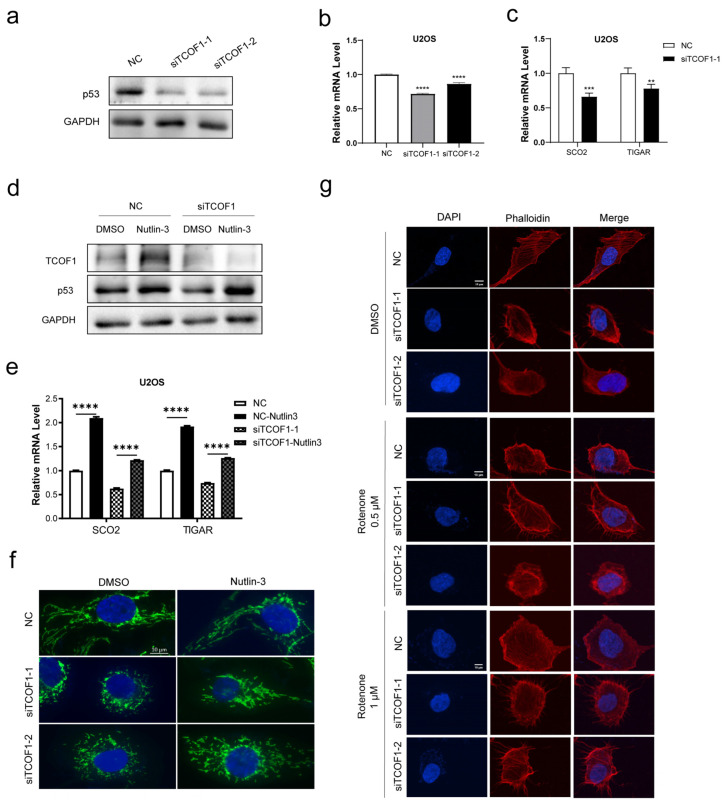
Deficiency of TCOF1 downregulates p53. (**a**) Western blotting analysis was performed to verify p53 protein expression levels in U2OS cells following TCOF1 deficiency. (**b**) RT-qPCR was used to validate p53 mRNA levels in U2OS cells after TCOF1 knockdown. (**c**) RT-qPCR analysis of SCO2 and TIGAR mRNA levels in U2OS cells after TCOF1 knockdown. (**d**) Western blotting of U2OS cells transfected with DMSO or Nutlin-3 and treated with NC (negative control) or siTCOF1 for 3 d at the same time. (**e**) The mRNA levels of SCO2 and TIGAR in (**d**). were detected by RT-qPCR. (**f**) Immunofluorescence staining revealed changes in mitochondrial morphology following TCOF1 knockdown and Nutlin-3 treatment in U2OS cells (100×). Scale bars: 10 μm. (**g**) Changes in F-actin morphology observed after 48 h treatment with 0.5 μM or 1 μM rotenone, respectively (100×). Scale bars: 10 μm. All values are the mean ± SEM of three independent experiments. The unpaired Student’s two-tailed *t*-test was used to determine the statistical significance (** *p* < 0.01, *** *p* < 0.001 and **** *p* < 0.0001).

p53 deficiency leads to reduced mitochondrial DNA (mtDNA) levels, impaired mitochondrial quality, and disruption of intracellular reactive oxygen species (ROS) homeostasis. This establishes p53’s critical role in maintaining mtDNA copy number and mitochondrial ROS homeostasis, confirming it as a key protein influencing mitochondrial function [39]. In the human osteosarcoma cell line U2OS, TCOF1 deficiency led to a consistent reduction in both p53 protein and mRNA levels (Figure 4a,b). It is suggested that TCOF1 may be involved in regulating the expression homeostasis of p53. However, the specific molecular regulatory mechanisms underlying this relationship remain to be further elucidated.

p53 can regulate mitochondrial respiratory activity [40]. Under normal conditions, p53 contributes to the maintenance of mitochondrial respiration by transcriptionally activating SCO2. When subjected to stress stimuli, p53 participates in mitochondrial repair or degradation processes through distinct mechanisms [26,41,42]. SCO2, as the key link connecting p53 to mitochondrial respiration, provides a potential theoretical framework for explaining the Warburg effect while offering novel insights into how p53 influences cellular aging and metabolic processes. The synthesis of SCO2 serves as a downstream mediator of p53’s regulatory role in mitochondrial respiration and is crucial for modulating cyclooxygenase (COX) complexes [26]. TIGAR (TP53-induced glycolysis and apoptosis regulator) is a target gene of p53, regulating mitochondrial respiration. p53 can activate TIGAR transcription and synthesis by binding to the promoter region of the TIGAR gene [43,44]. To this end, we detected that knocking down TCOF1 led to decreased mRNA levels of SCO2 and TIGAR (Figure 4c).

Murine double minute 2 (MDM2) functions primarily as a negative regulator of the p53 protein, maintaining p53 homeostasis within cells through multiple mechanisms to prevent excessive p53 activation that could cause cellular damage [45]. Nutlin-3, a small-molecule inhibitor specifically targeting the p53-MDM2 interaction, possesses a unique mechanism of action. It precisely interferes with the interaction between p53 and MDM2, thereby restoring the p53-mediated tumor suppression pathway. More importantly, while performing this function, Nutlin-3 produces minimal cytotoxicity and side effects, effectively enhancing cancer cell growth arrest and apoptosis [46]. Previous studies have demonstrated that Nutlin-3 significantly modulates and activates the p53 pathway [47]. To further investigate the link between p53 depletion and mitochondria, we treated TCOF1-deficient U2OS cells with Nutlin-3. The results showed that p53 protein levels, which were reduced due to TCOF1 deficiency, were markedly restored by Nutlin-3 treatment (Figure 4d). Concurrently, we assessed mRNA levels of SCO2 and TIGAR. Co-treatment with Nutlin-3 in TCOF1-deficient cells elevated mRNA expression of both SCO2 and TIGAR (Figure 4e). We conducted immunofluorescence experiments to observe mitochondrial morphological changes. Compared to the TCOF1 deficiency group without Nutlin-3, the TCOF1 deficiency group treated with Nutlin-3 exhibited significant alterations in mitochondrial morphology, characterized by a marked increase in tubular structures (Figure 4f). Rotenone, an inhibitor of mitochondrial electron transport chain complex I, causes mitochondrial DNA damage [48]. Administration of rotenone to *Drosophila melanogaster* induces mitochondrial superoxide production, which is associated with impaired locomotor function [49]. To further demonstrate that mitochondrial dysfunction contributes to F-actin imbalance, we treated cells with rotenone for 48 h. Results showed that rotenone reduced the polarity of cellular F-actin (Figure 4g), consistent with the morphological effects of TCOF1 deficiency on F-actin.

### 3.5. TCOF1 Maintains F-Actin Stability by Regulating p53

**Figure 5:**
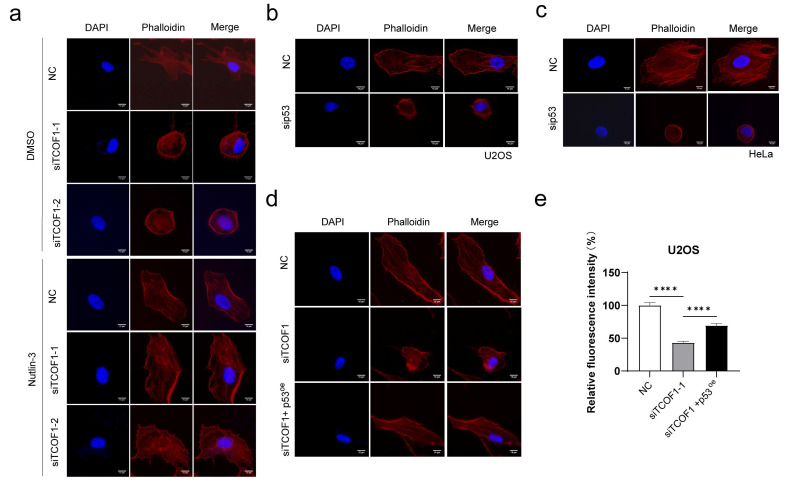
TCOF1 regulates p53 to maintain F-actin stability. (**a**) U2OS cells were transfected with NC (negative control), siTCOF1-1, or siTCOF1-2. After 24 h, DMSO or 10 μM Nutlin-3 was added to each group. Following 48 h of incubation, phalloidin staining was performed. (**b**) U2OS cells were transfected with NC and sip53, respectively, for phalloidin staining. Scale bars: 10 μm. (**c**) HeLa cells were transfected with NC and sip53 for phalloidin staining. Scale bars: 10 μm. (**d**) U2OS cells were transfected with NC, siTCOF1, siTCOF1 and p53^oe^ (overexpression), respectively, for phalloidin staining experiments. Changes in F-actin morphology were observed using a 100× objective lens. DAPI was used for nuclear staining (blue), phalloidin for actin staining (red), and Merge represents the overlay of nuclear and actin staining images. Scale bars: 10 μm. (**e**) Quantification of panel (**d**). All values are the mean ± SEM of three independent experiments. The unpaired Student’s two-tailed *t*-test was used to determine the statistical significance (**** *p* < 0.0001).

TCOF1 deficiency leads to decreased p53 protein levels, causing increased mitochondrial fragmentation and subsequent disruption of F-actin assembly, resulting in diminished cellular polarity. To further demonstrate that TCOF1 deficiency-induced p53 reduction is the cause of F-actin polarity loss, we detected changes in F-actin morphology by upregulating p53 protein expression using Nutlin-3. Through phalloidin staining experiments, we observed that TCOF1 deficiency resulted in disorganized F-actin cytoskeletal structures. Nutlin-3 partially reversed the cytoskeletal disruption caused by TCOF1 deficiency by activating p53 (Figure 5a). To further validate the pivotal role of p53 in this process, we individually knocked down p53 in U2OS cells and observed the structure of F-actin. We found that this similarly inhibited F-actin assembly and reduced cell polarity (Figure 5b). Phalloidin staining shows that p53 knockdown disrupts F-actin assembly in HeLa cells (Figure 5c). Furthermore, co-overexpressing p53 in TCOF1-deficient cells partially reversed the disruption of the F-actin cytoskeleton caused by TCOF1 deficiency (Figure 5d,e).

## 4. Discussion

The nucleolar phosphoprotein encoded by TCOF1 plays a crucial role in ribosomal biogenesis and cell cycle regulation, with its mutations closely associated with Treacher Collins Syndrome (TCS) [4]. As an autosomal dominant disorder, the core pathogenic mechanism of TCS stems from embryonic neural crest cell dysfunction caused by TCOF1 haploinsufficiency. TCOF1 deficiency significantly inhibits mature ribosome production in neuroepithelial and neural crest cells, triggering nucleolar stress and p53-mediated apoptosis [50]. As “seed cells” for craniofacial development, impaired proliferation and migration defects in neural crest cells directly cause abnormal differentiation of the first and second pharyngeal arches. This ultimately manifests as characteristic craniofacial malformations such as mandibular hypoplasia, external auditory canal atresia, and zygomatic bone absence [51]. TCOF1, as a nuclear factor, regulates transcriptional processes within the nucleolus by interacting with the upstream binding factor (UBF) [52]. TCOF1 is an essential molecule for the normal rDNA transcription within the nucleolus [1] and for ribosomal biogenesis and modification, thereby exerting a significant regulatory influence on global protein translation [53]. TCOF1 also ameliorates mitochondrial dysfunction in senescent cells by enhancing the transcription of nuclear-encoded mitochondrial proteins, thereby preserving mitochondrial homeostasis [11].

However, the precise mechanisms by which TCOF1 regulates mitochondrial function and cytoskeletal dynamics remain unclear. Our study reveals that TCOF1 deficiency significantly inhibits tumor cell migration, which depends on the dynamic reorganization of the cytoskeleton. The formation and assembly of F-actin are crucial for maintaining cell morphology and driving pseudopod extension. F-actin directly participates in directed cell movement through stress fibers and filopodia formed by polymerization, and structural abnormalities can impair cell motility [54]. In breast cancer cells, the marked disruption of the F-actin cytoskeleton results in a significant reduction in the cells’ migration and invasive capabilities [55,56]. In hepatocellular carcinoma (HCC) cells, TCOF1 deficiency similarly effectively inhibits their migration and invasion processes [57]. In our study, TCOF1 deficiency also reduced the migratory capacity of U2OS cells, indicating that TCOF1 influences the migratory ability of cancer cells. Abnormalities in the reciprocal regulation between mitochondria and the actin cytoskeleton directly disrupt cellular motility [58]. In this study, TCOF1 deficiency disrupted F-actin formation, potentially destabilizing cytoskeletal dynamics and thereby inhibiting tumor cell migration. This suggests that TCOF1 may serve as a critical molecular target for tumor metastasis by regulating F-actin homeostasis. Specific deficiency of the Pol I subunits Polr1a and Polr1c, along with the associated factor Tcof1, in neural crest cells (NCCs) of mice autonomously reduces rRNA synthesis; this leads to the accumulation of p53 protein, resulting in NCC cell apoptosis and craniofacial abnormalities [5]. CXCR5 precisely regulates neuronal polarity development and migration during embryogenesis by maintaining F-actin homeostasis. Disruption of this pathway leads to defects in neuronal polarity establishment and correlates with the pathological mechanisms of neurological disorders [59].

Further studies revealed that TCOF1 deficiency leads to impaired mitochondrial function, leading to aberrant F-actin assembly. Following TCOF1 knockout, mitochondria exhibited pronounced perinuclear aggregation and increased mitochondrial mass, suggesting TCOF1 may participate in regulating mitochondrial homeostasis. Mitochondria serve as cellular powerhouses, and their dysfunction directly impacts ATP synthesis efficiency. Reduced ATP production compromises the energy supply required for actin polymerization, leading to impaired microfilament assembly [31]. In this study, TCOF1 deficiency may impair mitochondrial function by disrupting the physical coupling between F-actin and mitochondria. Rotenone treatment disrupts F-actin assembly (Figure 4g), which is consistent with a previous report [60]. The resulting decrease in ATP production further weakens tumor cell migration capacity. Conversely, dysregulation of microfilaments disrupts mitochondrial function, thereby ultimately attenuating the migratory capacity of tumor cells. For instance, in breast cancer cells, F-actin depolymerization significantly decreases mitochondrial ATP generation efficiency, thereby inhibiting cellular motility [61].

This study also revealed that, beyond indirectly affecting mitochondrial function via F-actin, TCOF1 deficiency impacts mitochondrial function by downregulating p53 expression. The absence of TCOF1 significantly reduces p53 expression levels, and p53 serves as a crucial regulator of mitochondrial morphology and function. As a classic tumor suppressor, p53 not only participates in cell cycle regulation and apoptosis induction but also maintains mitochondrial respiratory chain integrity by transcriptionally activating mitochondrial-associated genes. Research indicates that p53 promotes mitochondrial elongation and maintains network stability by increasing phosphorylation, thereby inhibiting its translocation to mitochondria and regulating mitochondrial dynamics [62]. Downregulation of p53, a key regulator of mitochondrial fusion and fission, leads to excessive mitochondrial fission [63]. Similarly, TCOF1 depletion leads to the downregulation of p53, resulting in mitochondrial fission (Figure 4a,b,d). Abnormal activation of p53 may further deteriorate mitochondrial function by transcriptionally suppressing genes involved in mitochondrial biogenesis [64]. In human osteosarcoma U2OS cells, TCOF1 knockdown significantly reduced mRNA levels of p53, subsequently leading to decreased mRNA levels of the mitochondria-associated genes SCO2 and TIGAR downstream of p53, reflecting an imbalance in mitochondrial dynamics. We employed Nutlin-3 to activate the p53 pathway, validating p53 as a downstream effector molecule in TCOF1’s regulation of mitochondrial homeostasis and F-actin dynamics, thereby mediating tumor cell migration. Nutlin-3 treatment partially reversed TCOF1 knockdown-induced mitochondrial fragmentation, increased tubular structures, and partially ameliorated F-actin disorganization. Consequently, TCOF1 may influence mitochondrial function via p53-dependent pathways.

**Figure 6:**
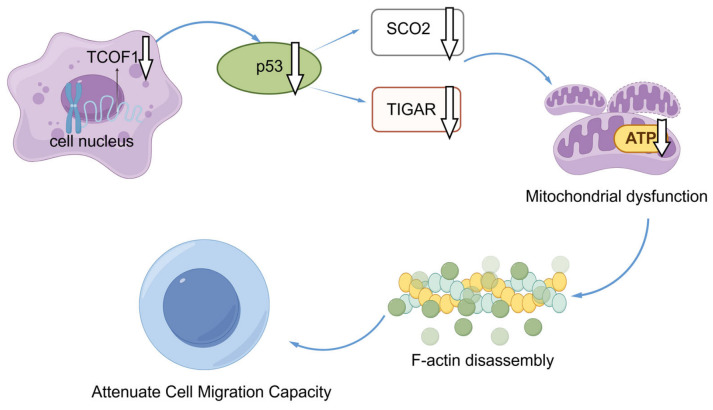
Schematic of the mechanism by which TCOF1 deficiency affects mitochondrial structure and function. White downward arrows indicate downregulation. This schematic was created using Figdraw 2.0 (https://www.figdraw.com, accessed on 3 February 2026).

This study establishes TCOF1 as a critical regulator of tumor cell migration by coordinating cytoskeletal dynamics and cellular energy metabolism through its dual control of F-actin assembly and mitochondrial function (Figure 6). These findings provide new molecular mechanisms for understanding TCOF1’s role in tumor progression and position it as a potential therapeutic target for suppressing metastasis. However, this study primarily conducted relevant experiments in U2OS cells. Although the results of TCOF1 knockdown in HeLa cells (Figure 2f) align with the trend observed in U2OS cells, the findings remain limited to a small number of cell lines. The universality of these conclusions requires further validation across a broader range of tumor cell lines.

## Figures and Tables

**Table 1:** siRNA sequences.

siRNA	Sequence (5′→3′)
NC (negative control)	5′-UUCUCCGAACGUGUCACGUdTdT-3′
siTCOF1-1	5′-GGAAUCAGAUAGUGAGGAAdTdT-3′
siTCOF1-2	5′-GAGGAUUCUUCAAGCAGUGAGGAAUdTdT-3′

**Table 2:** Primer sequences.

Primers	Sequences
TCOF1-forward primer	5′-CCCCAGAACAGTACCGTCTT-3′
TCOF1-reverse primer	5′-GCTCTGATCTGGTGGGTCTT-3′
GAPDH-forward primer	5′-AGCCACATCGCTCAGACAC-3′
GAPDH-reverse primer	5′-GCCCAATACGACCAAATCC-3′
β-actin-forward primer	5′-TCCCTGGAGAAGAGCTACGA-3′
β-actin-reverse primer	5′-AGCACTGTGTTGGCGTACAG-3′
MFN1-forward primer	5′-GAGGTGCTATCTCGGAGACAC-3′
MFN1-reverse primer	5′-GCCAATCCCACTAGGGAGAAC-3′
MFN2-forward primer	5′-CACATGGAGCGTTGTACCAG-3′
MFN2-reverse primer	5′-TTGAGCACCTCCTTAGCAGAC-3′
OPA1-forward primer	5′-AGTAGAGGTTGCTTGGGAGAC-3′
OPA1-reverse primer	5′-TGTCATCATGCTCTTTCCCT-3′
Fis1-forward primer	5′-CATCGTGCTGCTGGAGAGC-3′
Fis1-reverse primer	5′-GCAGAGAGCAGGTGAGGCTG-3′
NDUFS1-forward primer	5′-TTAGCAAATCACCCATTGGACTG-3′
NDUFS1-reverse primer	5′-CCCCTCTAAAAATCGGCTCCTA-3′
SDHA-forward primer	5′-CAGCATGTGTTACCAAGCTGT-3′
SDHA-reverse primer	5′-GGTGTCGTAGAAATGCCACCT-3′
UQCRC1-forward primer	5′-GGGAGTGTGGATTGATGTTGG-3′
UQCRC1-reverse primer	5′-TGTTCCCTTGAAAGCCAGATG-3′
ATP5B-forward primer	5′-GGTGAGAGGACCCGTGAAG-3′
ATP5B-reverse primer	5′-CGCTACCTTAGAGGTGGCAT-3′

## Data Availability

The original contributions presented in this study are included in the article/Supplementary Materials. Further inquiries can be directed to the corresponding authors.

## Supplementary Materials

The following supporting information can be downloaded at: https://www.mdpi.com/article/10.3390/cimb48050447/s1.
