# Supplementary material for: TCOF1 Regulates Tumor Cell Migration Through p53-Dependent Mitochondrial Homeostasis and F-Actin Dynamics
Source: Curr Issues Mol Biol. 2026 Apr 24;48(5):447. doi: 10.3390/cimb48050447 (PMC13204259; doi:10.3390/cimb48050447)
Supplement: Supplementary file 1 [file cimb-48-00447-s001.zip › Supplementary Figure 1.pdf]

Figure S1

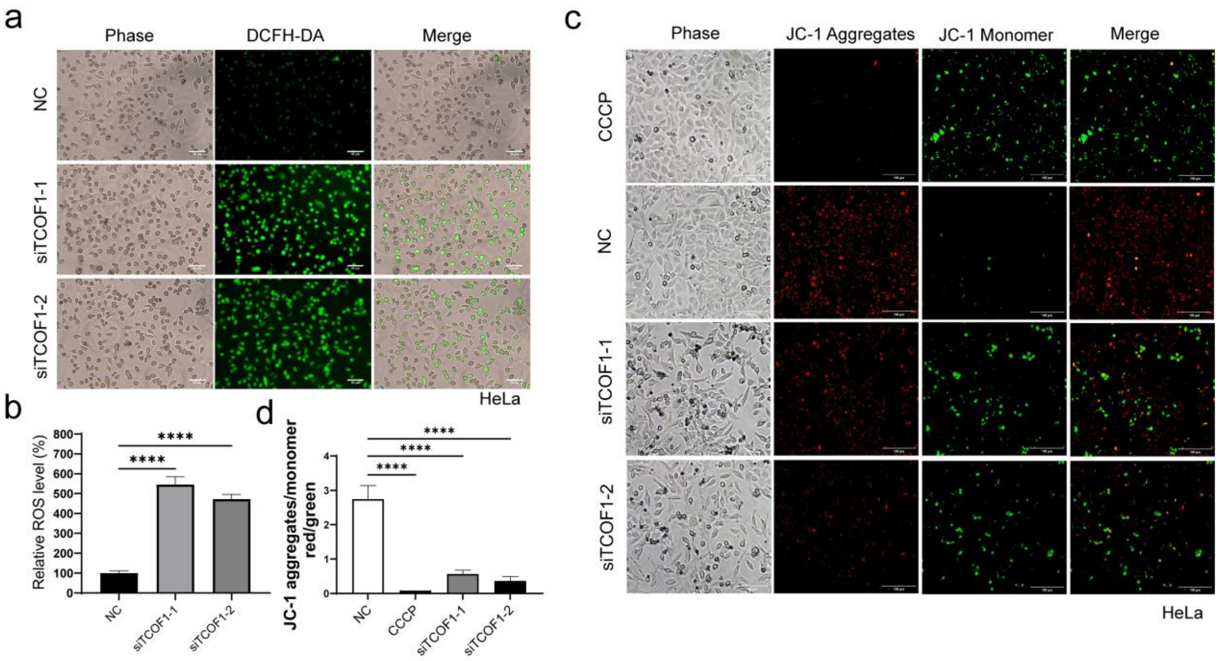

Figure S1: (a) Fluorescence images of reactive oxygen species (ROS) in HeLa cells loaded with DCFH-DA probe under NC, siTCOF1-1, and siTCOF1-2 conditions. (b) Quantification of panel (a). (c) Fluorescence microscopy images of JC-1 staining in indicated groups (Scale bar, 100 μm). CCCP-treated cells served as a positive control for mitochondrial depolarization. HeLa cells were transfected with negative control or siTCOF1-1/2 siRNA, respectively. Red fluorescence (JC-1 Aggregates) indicates intact ΔΨm, whereas green fluorescence (JC-1 Monomer) indicates loss of ΔΨm. Scale bars：10 μm. (d) Quantification of panel (c).
